# Quality of life and social engagement of alcohol abstainers and users among older adults in South Africa

**DOI:** 10.1186/1471-2458-14-316

**Published:** 2014-04-05

**Authors:** Priscilla Martinez, Lars Lien, Anne Landheim, Paul Kowal, Thomas Clausen

**Affiliations:** 1 Norwegian Center for Addiction Research, University of Oslo, Oslo, Norway; 2 Alcohol Research Group, UC Berkeley, School of Public Health, 6475 Christie Ave, Suite 400, Emeryville, CA 94608-1010, USA; 3 Center of Excellence for Substance Abuse and Addiction, Innland Hospital Trust, Ottestad, Norway; 4 Department of Research, Innland Hospital Trust, Ottestad, Norway; 5 Research Centre for Gender, Health and Ageing, University of Newcastle, Newcastle, Australia

**Keywords:** South Africa, Older adults, Drinking pattern, Quality of life, Social engagement

## Abstract

**Background:**

The literature from developed countries suggests a relationship between alcohol use and quality of life and social engagement, where harmful drinkers have lower quality of life and less social engagement. Despite the high rates of harmful alcohol use in South Africa, little is known about the association between drinking pattern and quality of life and social engagement in this context. We aimed to determine if quality of life and social engagement varied across different drinking patterns among older South African adults, contributed to drinking pattern when controlling for socio-demographic factors, and varied differentially between genders.

**Methods:**

This is a secondary analysis of data from the Survey on Global Ageing and Adult Health (SAGE). Alcohol use was measured as self-reports of use over the previous seven days, and we constructed gender-specific alcohol variables. The WHO Quality of Life-scale was used to measure quality of life, and social engagement was measured by frequency of participation in social activities. We used ANOVA to observe differences in quality of life and social engagement scores across drinking patterns, and regression models were used to identify factors independently associated with drinking pattern.

**Results:**

There were 2572 (84.4%) lifetime abstainers, and 475 (15.6%) persons who had a drink in the last 7 days. In bivariate analysis, quality of life was lowest among at risk drinking men compared to abstainers (OR = 0.21, p = 0.02), although this association was not significant in adjusted analysis. Social engagement did not vary statistically significantly across the drinking patterns in the total sample or within gender.

**Conclusions:**

Quality of life and social engagement were not independently associated with drinking pattern among older adults in South Africa in this sample. In order to better understand their alcohol use, further exploratory research is warranted to identify other potentially relevant subjective factors of alcohol use among older adults in South Africa.

## Background

Alcohol use is an important contributor to the burden of non-communicable diseases in the Republic of South Africa [1]. Moreover, a recent population-based study from South Africa report current and harmful alcohol use is on the rise [2]. Among older adults, the harmful use of alcohol can impact disease symptom severity and progression of common chronic illnesses, in addition to negatively affecting personal relationships, social engagement and overall quality of life [3,4].

Studies from developed countries have identified a linear or inverse J-shaped relationship between quality of life and alcohol use, such that at the higher levels of alcohol use, including persons diagnosed with alcohol use disorders, quality of life is lower compared to moderate or low risk users and alcohol abstainers [5,6]. Among problem drinkers in treatment, the literature suggests the measurement of quality of life can both be a motivational tool for behavior change and a measure of treatment efficacy [7]. Less is known about how quality of life predicts alcohol use, although a causal relationship in this direction is theoretically plausible.

Social engagement encompasses activities such as attending religious services, participating in clubs or group activities, and spending time with friends and family. It is related to alcohol use and problem drinking in that it can reinforce drinking behaviors, or be consequent to established alcohol using patterns [8,9]. Moreover, substantial evidence shows high alcohol consumption is associated with divorce [10], poor family relations [11] and problems in the work place [12].

Self-reported lifetime abstention from alcohol is common among the general older adult population in South Africa, yet risky drinking patterns are common among those who do imbibe [13,14]. These patterns are frequent among both South African men and women, where the high rates of harmful drinking among women, especially women of child-bearing age, has been a cause for concern for mother-to-child HIV transmission and Fetal Alcohol Syndrome [15]. Indeed, South Africa has one of the highest rates of Fetal Alcohol Syndrome worldwide [16]. Other health behaviors, such as smoking, and socio-cultural factors, such as marital status and ethnicity, have been found to be associated with risky drinking behaviors among older adults in South Africa, with considerable differences between the sexes [17]. Religious affiliation is a strong predictor of drinking behavior in South Africa among both genders, where studies consistently observe that Christians imbibe and Muslims abstain [13,18].

There is scant information on the quality of life and social engagement of abstainers and drinkers among older adults in South Africa, how these aspects may vary across different drinking patterns and what relationship they have to drinking behavior in the context of other socio-cultural determinants. Efforts are underway to develop alcohol policies aimed at the general population to reduce alcohol-related harm in South Africa, and older adults are a growing segment of the general public. Thus, it will benefit national policy makers and community-based program planners to better meet the physical and mental health needs of this important group to have evidence about whether drinking pattern is associated with quality of life and social engagement, and therefore if these measures could be used as motivational tools or treatment outcome measures. This paper addresses the following research questions: (a) How does subjective quality of life and self-reported social engagement differ between lifetime abstainers, ‘low risk’ and ‘at risk’ drinkers stratified by gender?; (b) Does quality of life and social engagement contribute to drinking pattern assignment among men and women separately when controlling for socio-cultural factors and smoking?; and, (c) Are there differences at the item level of the quality of life and social engagement scales between drinking patterns, stratified by gender?

## Methods

### Data collection

Data were collected through Wave 1 of the World Health Organization’s Study on global AGEing and Adult Health (SAGE). SAGE was implemented in 2007–2010 in six countries, including South Africa. SAGE collected individual level data from nationally representative household samples of older adults (aged 50-plus years) using a multi-stage cluster sampling design, and included a smaller sample of younger adults (aged 18–49 years) for comparison purposes. We did not use the sample of younger adults in this analysis. Standardized questionnaires were used to collect data, including measures of health, behavioral risk factors, quality of life and social engagement. SAGE protocols and procedures were approved by the WHO Ethical Review Committee and ethics committees in each participating country, and informed consent was obtained from all participants. SAGE is described in detail elsewhere [19].

### Sample

The sample of adults aged 50-plus included 3666 participants in South Africa, 2108 (56.1%) of whom were women. This analysis focused on lifetime abstainers and current alcohol consumers; we therefore did not include the 506 people who reported ever having had a drink but who did not consume alcohol in the last seven days. A total of 97 respondents had missing data on key variables including alcohol consumption, quality of life or social engagement, and were thus excluded. The final sample size was comprised of 3047 persons, where 1811 (59.4%) were women.

In this final sample, approximately 10% of the ethnicity and religion variables were missing. Given the relevance of these factors to drinking patterns and the potential for introducing biased estimates if cases were excluded, multiple imputation was used to address the missing values. Data were missing at random according to correlation matrices between missingness for ethnicity and religion and other covariate values, and logistic regression models assessing the association between missingness and the value of the missing variables controlling for other covariates [20]. Ten data sets were imputed using multinomial regression models for ethnicity and religion including all independent, dependent and structural sampling variables (that is, strata, probability sampling unit, person weights) in the model. The imputed datasets were then used for all the regression models, employing the survey (svy) and multiple imputation (mi, mi svyset) command structures in STATA version 12.0.

### Variables

#### Alcohol measures

Alcohol measures followed the WHO STEPS guidelines [21], with lifetime abstinence based on the response to an initial question, “Have you ever consumed a drink that contains alcohol?” If the respondent responded positively, they were asked about the number of standard drinks consumed on each day in the preceding seven days. Lifetime abstainers were defined by a negative response to the question regarding ever consuming alcohol. Two mutually exclusive drinking categories were generated: ‘low risk’ drinkers, defined as only 1–2 drinks per day over the last 7 days and no more than 7 in total for women and 14 in total for men; and, ‘at risk’ drinkers, defined as either four or more drinks on any one day in the previous seven days for women and five or more drinks for men, or eight drinks in total for women and 15 for men over the previous seven days. Men and women who consumed 3 drinks per day and no more than 7 in total over the previous 7 days for women and 14 in total for men were not included in the “low risk” and “at risk” categories and were thus not included in the analysis. These definitions are based on the guidelines from the US National Institute of Alcohol Abuse and Alcoholism (NIAAA) for adult men and women [22]. A country-specific showcard was used with pictures to illustrate to respondents what was meant by a “standard drink”, including alcohol equivalents.

#### Quality of life

The SAGE Survey used the 8-item WHO Quality of Life scale (WHOQoL-8) to measure subjective quality of life. The individual items queried about the level of satisfaction one felt in regards to various aspects of one’s life, such as money, health and relationships. The WHOQoL has been used widely in many settings, including countries in Africa [23]. The responses are based on a 5-point Likert scale, where “1” indicated high satisfaction and “5” indicated low satisfaction. A quality of life index score was calculated by reversing the numerical codes and summing the individual responses such that higher responses indicated a higher quality of life. Scores ranged between a minimum of 8 and a maximum of 40, and were normally distributed. We also dichotomized the individual item responses into “high” and “low” by collapsing the two highest positive responses “very satisfied/completely” and “satisfied/mostly” into a “high” category, and the three lowest negative responses “neither satisfied nor dissatisfied/moderately”, “dissatisfied/a little” and “very dissatisfied/none at all” into a “low” category. In this sample, the Cronbach’s alpha of the entire scale was 0.87.

#### Social engagement

The measures of social engagement included nine questions about how often participants engaged in a particular social activity in the last 12 months, including attendance at a public meeting discussing local affairs, personally meeting a community leader, attending any group meeting (club, union, society, organization), working with other people in the neighborhood to improve or fix something, having friends visit their home, being in the home of or hosting someone from a different neighborhood, socializing with coworkers outside work, attending religious services, and leaving the house to attend meetings, activities, visit family or friends. The subject responded to each item according to the response options “never”, “once or twice per year”, “once or twice per month”, “once or twice per week” or “daily”, coded numerically from 1 (“never”) to 5 (“daily”). We calculated a social engagement index score by summing the corresponding responses, where higher scores indicated higher social engagement. Scores ranged from a minimum of nine to a maximum of 45, and were normally distributed. We also dichotomized responses into “regularly” vs. “irregularly”, where “regular” participation included monthly, weekly and daily responses, and “irregular” included annual (once or twice per year) or no participation (never). In this sample, the Cronbach’s alpha of the entire scale was 0.76.

#### Other measures

The question “do you currently use any tobacco products?” was used to define current smokers as those who responded “yes, daily” and “yes, but not daily”. Any chronic illness in the last 12 months was based on self-reported symptoms in the previous 12 months for arthritis, angina, diabetes, chronic lung disease and asthma.

### Statistical analysis

All SAGE Wave 1 data were weighted, with post-stratification adjustments for age and sex based on UN population estimates (UN Pop Div World Pop Prospects: The 2010 Revision. 2011). Descriptive statistics are presented as weighted proportions for categorical variables, weighted means with standard errors for continuous variables, and stratified by sex. All sample sizes are un-weighted raw numbers. Proportions for ethnicity and religion are based on the imputed datasets. Proportions for lifetime abstainers are presented out of the total sample, and low- and at-risk drinkers out of respondents who reported drinking in the last seven days unless otherwise noted.

Analysis of variance was used to test for differences in means of quality of life and social engagement scores across the drinking patterns within gender and overall. Multinomial logistic regression was used to test for differences between lifetime abstainers and low- and at-risk drinkers on quality of life or social engagement, with lifetime abstainers as the referent group. Chi-square test of independence was used for analyses of categorical variables and drinking patterns, and an F statistic was reported that accounts for survey design effects and is equivalent to the chi-square [24]. We considered a p-value of ≤0.05 to be statistically significant.

Multinomial logistic regression models were fitted to estimate the independent contribution of quality of life and social engagement scores on drinking pattern assignment. Models were adjusted for covariates significantly associated with drinking pattern in bivariate analysis, including age, ethnicity, marital status, ever educated, religion, any chronic illness in the last 12 months and current smoking status. Adjusted odds ratios and p-values are reported.

To further explore the relationship between quality of life and social engagement and drinking patterns, the frequency of the dichotomized individual items of each index in each drinking category by gender was calculated and tested for differences across the drinking patterns using the chi-square test of independence. To compare drinking patterns and responses at the individual item level, we fit bivariate logistic regression models with the dichotomous item response as the dependent variable and drinking pattern as the independent variable. Lifetime abstainers were the referent group. Only data for the items that showed variation across the drinking patterns are presented.

## Results

There were 2572 (84.4%) lifetime abstainers, and 475 (15.6%) persons who had a drink in the previous seven days. Of these, 308 (64.8%) were low risk drinkers and 167 (35.2%) were at risk drinkers. The mean quality of life and social engagement scores were 27.4 (SE = 0.19) and 21.2 (SE = 0.22), respectively. Demographics and quality of life and social engagement scores by drinking pattern for men and women are presented in Table 1.

**Table 1:** Demographics, quality of life and social engagement scores by drinking pattern among 50+ South African men and women

	**Lifetime abstainers Mean (SE) or Proportion**			**Low risk drinkers Mean (SE) or Proportion**			**At risk drinkers Mean (SE) or Proportion**			**Statistic**		
	**Women n=1643**	**Men n=929**	**Total n=2572**	**Women n=100**	**Men N=208**	**Total N=308**	**Women n=68**	**Men N=99**	**Total n=167**	**Within women**	**Within men**	**Within totals**
Age	62.1 (0.40)	61.4 (0.56)	61.9 (0.36)	59.9 (0.97)	59.2 (0.80)	59.4 (0.60)	59.3 (1.92)	59.4 (0.99)	59.4 (1.02)	F=2.50, p=0.08	F=3.41, p<.05	F=6.9, p≤.001
Ethnicity												
African/Black	73.8	70.2	72.1	72.6	65.4	66.8	64.7	83.6	75.8			
White	7.8	10.0	8.7	14.3	12.4	13.2	11.1	7.0	8.7			
Coloured	13.8	12.7	13.5	10.2	19.7	17.4	23.2	7.4	13.9			
Indian/Asian	4.5	7.2	5.7	2.8	2.5	2.6	1.1	2.0	1.6	F=0.31, p=0.74	F=2.10, p=0.05	F=1.98, p=0.07
Married	36.3	80.6	53.5	45.0	73.2	65.7	42.5	73.4	60.4	F=0.85, p=0.43	F=1.33, p<0.05	F = 2.27, p=0.11
Ever schooled	77.6	77.7	77.6	57.9	83.3	76.5	73.1	75.9	74.7	F=3.62, p<0.05	F=0.87, p=0.42	F=0.16, p=0.85
Religion												
None/other	6.5	9.9	7.8	6.6	11.5	10.2	11.9	15.6	14.0			
Christianity	88.1	86.2	87.4	91.6	83.5	85.7	83.0	76.2	79.1			
Islam	3.0	3.0	3.0	0	0	0	0	1.3	0.7			
Primal indigenous	2.3	1.0	1.8	1.8	4.9	4.1	5.1	6.9	6.2	F=13.0, p<0.01	F=4.00, p≤.001	F=4.91, p≤.001
Currently working	23.4	39.0	29.5	24.1	41.9	37.2	17.4	42.0	31.5	F=0.36, p=0.70	F=0.10, p=0.90	F=0.87, p=0.42
Rural setting	35.9	32.4	34.6	29.1	34.6	33.1	47.9	21.7	32.9	F=1.16, p=0.32	F=1.52, p=0.22	F=0.05, p=0.95
Chronic illness <12 mo.	50.0	41.4	46.7	46.0	50.3	49.2	39.3	58.5	50.3	F=0.65, p=0.52	F=3.24, p<0.05	F=0.26, p=0.77
Current smoker	14.9	14.2	14.6	40.6	68.5	61.0	64.6	68.6	66.9	F=18.3, p≤.001	F=51.5, p<.001	F=80.4, p≤.001
Quality of life score	27.0 (0.26)	28.1 (0.23)	27.4 (0.21)	27.3 (0.68)	27.5 (0.77)	27.4 (0.61)	27.4 (1.04)	26.6 (0.64)	26.9 (0.58)	F=0.14, p=0.87	F=2.98, p=0.05	F=0.43, p=0.65
Social engagement score	20.9 (0.28)	21.9 (0.38)	21.3 (0.25)	19.8 (0.69)	21.1 (0.52)	20.8 (0.42)	20.5 (0.94)	21.8 (0.91)	21.3 (0.69)	F=1.20, p=0.30	F=0.60, p=0.55	F=0.57, p=0.56

### Quality of life and social engagement differences across drinking pattern stratified by gender

In bivariate analysis, quality of life varied statistically significantly among men, where at risk drinking men had the lowest score, and was significantly lower relative to lifetime abstainers (OR = 0.21, 95% CI 0.06, 0.81, p = 0.02). Among women, the mean quality of life score varied by only 0.4 points across the three drinking categories.

Social engagement did not vary significantly across the drinking patterns in the total sample or by sex (Table 1). Among the whole sample, social engagement was lowest among low risk drinkers although the difference across the drinking patterns was not statistically significant. This pattern of lower but not statistically significant differences in social engagement scores among low risk drinkers was also observed among both women and men.

### Contribution of quality of life to drinking pattern assignment controlling for socio-cultural factors and smoking

Given that quality of life among women and social engagement in the whole sample and by sex were not associated with drinking category in bivariate analysis, a multivariable model for quality of life and drinking pattern among men only was fitted. In multivariable analysis, adjusting for socio-demographics, chronic conditions and smoking status, quality of life among men was not significantly associated with being a low risk (OR = 1.00, 95% CI 0.93, 1.06) or at risk drinker (OR = 0.99, 95% CI 0.92, 1.06) relative to lifetime abstainers.

### Differences at the item level of quality of life and social engagement scales between drinking patterns by gender

There were no significant variations between the drinking categories on any quality of life items among women. For individual items on the social engagement index, low risk drinking women had the lowest proportion of regular attendance at community improvement activities (OR = 0.39, 95% CI 0.17, 0.85, p = 0.02) and public meetings (OR = 0.50, 95% CI 0.24, 1.04, p = 0.06) as compared to lifetime abstainers. At risk drinking women had the lowest proportion of regular attendance to religious services (OR = 0.23, 95% CI 0.09, 0.59, p = 0.002) compared to lifetime abstainers (Table 2).

**Table 2:** Selected quality of life and social engagement scale items by gender and drinking pattern for 50+ adults in South Africa

	**Women**			
	**Lifetime abstainers (%)**	**Low risk drinkers (%)**	**At risk drinkers (%)**	**Statistic**
**Social engagement items**				
Regular public meeting attendance	22.6	12.7	21.6	F=1.78, p=0.17
Regular community improvement activities	26.8	12.3	29.7	F=2.93, p=0.05
Regular religious activity attendance	82.7	78.2	52.5	F=4.84, p<0.01
	**Men**			
**Quality of life items**				
Satisfied with health	69.1	61.9	56.0	F=1.72, p=0.18
Satisfied with self	83.5	77.8	68.6	F=2.91, p=0.06
Satisfied with money	15.1	14.7	7.0	F=1.89, p=0.15
**Social engagement items**				
Regular club/group meeting attendance	41.2	27.9	39.8	F=2.57, p=0.08
Regular visits from friends	74.9	84.2	81.6	F=2.55, p=0.08
Regular visits to/from other neighborhoods	62.6	77.5	74.7	F=5.20, p<0.01
Regular religious activity attendance	82.8	63.6	49.2	F=11.52, p<0.001

Among men, at risk drinkers reported a significantly lower proportion of “high” satisfaction on quality of life items for health (OR = 0.57, 95% CI 0.30, 1.08, p = 0.08), oneself (OR = 0.43, 95% CI 0.21, 0.88, p = 0.02) and money (OR = 0.42, 95% CI 0.18, 1.01, p = 0.05) (Table 2) compared to lifetime abstainers. For individual items on the social engagement scale, we observed differences on regular attendance at club/group meetings, having friends over to one’s home, visiting or having been visited by someone from a different neighborhood, and attending religious activities. Low risk drinking men reported the lowest regular attendance at club/group meetings compared to lifetime abstainers (OR = 0.55, 95% CI 0.25, 0.92, p = 0.03). Conversely, they reported the highest proportion of regular visits from friends in their homes (OR = 1.79, 95% CI 1.02, 3.09, p = 0.04), and visiting or having been visited by someone from a different neighborhood (OR = 2.05, 95% CI 1.29, 3.34, p = 0.003). At risk drinking men reported the lowest proportion of regular attendance at religious activities compared to lifetime abstainers (OR = 0.20, 95% CI −0.44,0.23, p < 0.001).

## Discussion

This analysis observed no differences in social engagement between different types of drinking patterns among adults aged 50 and above in South Africa. Similarly, in the total sample and among women, we found no differences in quality of life between the different drinking patterns. Among men, quality of life was significantly lowest among at risk drinkers in bivariate analysis, but did not contribute to drinking pattern status when controlling for other covariates. Regular participation in religious activities was lowest among both at risk drinking men and women, while the other social engagement items associated with drinking pattern differed between the genders.

The observation of a lower quality of life among at risk drinking men is consistent with other population-based studies using the Wool scale [25] and other measures of quality of life [26], and among clinical samples of alcohol dependent males [27]. It supports other studies demonstrating diminished quality of life with increased alcohol consumption [28,29]. In contrast to this, however, is the finding of no difference in quality of life generally, among women across the drinking patterns and among men in adjusted analysis. Supporting the local validity of this finding is a recent study from South Africa among outpatients that reported no association between alcohol use disorders and health-related quality of life [30].

The few investigations of quality of life and drinking among older adults include positive findings between psychological distress and binge drinking among women, but not men [31], and a modest association between poorer psychological well-being and heavier drinking among a community sample of older adults [32]. Clearly more information is needed to better understand this relationship among older adults, particularly in an African context. If no association between quality of life and alcohol use is consistently observed in South Africa, this will inform local alcohol program developers and evaluators to consider quality of life as an outcome measure with limited value for alcohol prevention and intervention efforts.

Reports of a higher quality of life among moderate, current drinkers compared to former drinkers, abstainers or high consumers, suggests an inverse U-shaped relationship between quality of life and alcohol use [6,33,34]. While the differences in this study were not statistically significant, the trend was a linear decline with increasing alcohol use from lifetime abstainers to at risk drinkers among men, and virtually no change among women. This equivocally suggests a significant inverse U-shaped relationship between subjective quality of life and alcohol use is not currently relevant among older adults in this context. In other words, quality of life may not be a useful measure as a predictor or consequence of alcohol use among community dwelling South African adults over 50 years of age. There are several potential explanations for this observation of a lack of an association between quality of life and drinking pattern among older adults in South Africa. One potential explanation is that the development and recognition of negative consequences of heavy alcohol use among older adults in South Africa may not yet have fully manifested. The four-stage model of the cigarette epidemic indeed intends to describe the delay between the adoption of a risky health behavior (smoking) and its effect on health at the population level [35]. The anticipated increase in alcohol use in South Africa in particular and Africa in general may follow a similar trajectory, so that the negative social and psychological effects of heavy alcohol use may become apparent only at a later stage after a critical mass has adopted the behavior. A second potential explanation of the observation of no association between quality of life and drinking pattern is that the cultural environment moderates the local meaning of quality of life and alcohol use such that they are not related to one another, that is, among older, community-dwelling South African results, quality of life may indeed not be associated with alcohol consumption. A third possible explanation particular to this study is that the large number of lifetime abstainers and relatively small number of current, past week drinkers may have limited the study’s ability to observe an association. In short, the statistically insignificant findings between quality of life and alcohol use are potentially a real finding, or a methodological artifact. To address this uncertainty and test the veracity of these results, studies among current alcohol users with varying levels of use and which compare different measures of quality of life are warranted.

At risk drinking men and women reported the lowest regular participation in religious activities. There are at least two possible mechanisms for this association. One mechanism is that active participation in religious gatherings is an important factor in determining alcohol use through positive social reinforcement of moderate drinking behavior. Another mechanism is that those who develop harmful drinking patterns are selected out among those regular religious participants through the negative reinforcement of at risk drinking behavior [36]. Several studies have shown religious affiliation is strongly associated with alcohol consumption in several African states [13,18], and further longitudinal investigations are required to elucidate causal pathways. South African low-risk drinking men had the highest frequency of regularly engaging in activities with friends, and this is similar to results among older adults in the US showing social participation is associated with more alcohol consumption, and indicating drinking is undertaken for social reasons [37,38]. Our findings suggest drinking may be a component of the social activities of low risk drinking older South African men, potentially both through causal and consequent pathways.

The limitations of this study deserve mention. Self-reported measures for alcohol consumption are subject to under-reporting and desirable response bias, and may have contributed to heterogeneity within the drinking categories through misidentification of participants actual drinking habits. Further, the lack of a specific measure of alcohol use disorders or alcohol problems, such as a diagnostic instrument or a validated screener, may have reduced the accuracy of the drinking categories and limited our ability to detect any associations. Also, the small absolute numbers of low and at risk drinking women may have contributed to a limited ability to detect differences. Finally, the number of statistical tests run with the individual items of the quality of life and social engagement scales increases the likelihood of a Type I error.

## Conclusions

The associations between quality of life and social engagement with alcohol use previously observed in European countries and the US were not replicated in this study among older South African adults. The reasons for this divergence could be different “timing” in the epidemic of alcohol related harm across continents or cultural differences affecting the relationship between drinking patterns and how it relates to quality of life and social engagement. In contrast to other regions of the world, the utility of assessing quality of life as a motivational tool for people with alcohol use disorders or as a measure of treatment efficacy may be limited in South Africa. Further exploratory research is warranted to identify other potentially relevant subjective factors of alcohol use among older adults in South Africa in order to better understand the context, predictors and consequences of their alcohol use and to ensure fidelity between public health interventions and local needs.

## Competing interests

The authors declare that they have no competing interests.

## Authors’ contributions

PM, TC, and LL conceived and designed the study. PK collected data. PM conducted data analysis and wrote the first manuscript. All authors contributed to interpretation and critical revisions of the article. All authors had access to the data in the study, and can take responsibility for the integrity of the data and the accuracy of the data analysis, and edited and approved the final version.

## Pre-publication history

The pre-publication history for this paper can be accessed here:

http://www.biomedcentral.com/1471-2458/14/316/prepub
